# Effectiveness of endourological management of ureteral stenosis in kidney transplant patients: EAU-YAU kidney transplantation working group collaboration

**DOI:** 10.1007/s00345-023-04455-3

**Published:** 2023-06-20

**Authors:** Angelo Territo, Alejandra Bravo-Balado, Iulia Andras, Riccardo Campi, Alessio Pecoraro, Vital Hevia, Thomas Prudhomme, Michael Baboudjian, Andrea Gallioli, Paolo Verri, Mathieu Charbonnier, Romain Boissier, Alberto Breda

**Affiliations:** 1 Department of Urology, Fundació Puigvert and Autonomous University of Barcelona, Carrer de Cartagena 340, Fundació Puigvert, 08025 Barcelona, Spain; 2 Urology Department, Iuliu Hatieganu University of Medicine and Pharmacy, Cluj-Napoca-Napoca, Romania; 3 Unit of Urologic Robotic, Minimally-Invasive Surgery and Renal Transplantation, Careggi University Hospital, Florence, Italy; 4 Department of Experimental and Clinical Medicine, University of Florence, Florence, Italy; 5 Hospital Universitario Ramón y Cajal. Alcalá University. Instituto Ramón y Cajal de Investigación Sanitaria (IRYCIS), Madrid, Spain; 6 Department of Urology and Kidney Transplantation, Toulouse University Hospital, Toulouse, France; 7 Department of Urology and Kidney Transplantation, APHM, CHU de La Conception Hospital, 146, Boulevard Baille, 13005 Marseille, France

**Keywords:** Endourology, Kidney transplantation, Ureteral stenosis

## Abstract

**Purpose:**

Ureteral stenosis (US) in kidney transplant (KT) recipients is associated with poorer long-term graft survival. Surgical repair is the standard of care, and endoscopic treatment represents an alternative for stenosis < 3 cm. We aimed to determine the effectiveness and safety of endourological management of US in KT patients and predictors of failure.

**Methods:**

A retrospective multicenter study was conducted in four European referral centers, including all KT patients with US managed endoscopically between 2009 and 2021. Clinical success was defined as the absence of upper urinary tract catheterization, surgical repair or transplantectomy during follow-up.

**Results:**

A total of 44 patients were included. The median time to US onset was 3.5 months (IQR 1.9–10.8), the median length of stricture was 10 mm (IQR 7–20). Management of US involved balloon dilation and laser incision in 34 (79.1%) and 6 (13.9%) cases, respectively, while 2 (4.7%) received both. Clavien–Dindo complications were infrequent (10%); only one Clavien ≥ III complication was reported. Clinical success was 61% at last follow-up visit (median = 44.6 months). In the bivariate analysis, duckbill-shaped stenosis (vs. flat/concave) was associated with treatment success (RR = 0.39, *p* = 0.04, 95% CI 0.12–0.76), while late-onset stenosis (> 3 months post KT) with treatment failure (RR = 2.00, *p* = 0.02, 95% CI 1.01–3.95).

**Conclusions:**

Considering the acceptable long-term results and the safety of these procedures, we believe that the endoscopic treatment should be offered as a first-line therapy for selected KT patients with US. Those with a short and duckbill-shaped stenosis diagnosed within 3 months of KT seem to be the best candidates.

## Introduction

The incidence of ureteral stenosis (US) in kidney transplant (KT) patients is around 5%, and goes up to 10% at 5 years [1], representing the most common long-term urological complication [2]. It is negatively correlated with long-term renal graft survival [4]; consequently, timely diagnosis and treatment are mandatory. Ureteral stenosis typically becomes evident within the first 3 months after KT and is primarily caused by ureteral ischemia: loss of distal ureteral perfusion through graft explantation in the situation of an ureterovesical anastomosis or loss of the proximal perfusion of the native ureter in case of pyelo-ureteral anastomosis. Late stenosis is rare, and less common causes should be explored, such as vascular disease, fibrosis, infection, reactivation of BK virus or allograft rejection [5, 6]. Historically, the open surgical approach has been the main treatment option for these patients, especially in cases of complex, mid-third or proximal US [7, 8], given its association with increased renal graft survival [8]. Although the surgical reconstruction has gained new insights due to the possibility of robot-assisted US repair, even minimally invasive reconstructive approaches may have associated complications that are not negligible [9]. Given recent advances in endourological instrumentation, an endoscopic approach, using either percutaneous balloon dilatation or flexible antegrade ureteroscopy and holmium laser incision, has been proposed as a reasonable alternative for stenosis < 3 cm. To date, data on endoscopic treatment of US after KT are scarce and come from a limited number of single-center series. Factors that may predict outcomes of the endoscopic management are also not known, although they could improve patient selection for optimal results. Our objective was to determine the effectiveness and safety of the endourological management of US in KT patients in four European institutions and to determine predictive factors of clinical failure.

## Materials and methods

This retrospective, multicenter study was conducted in accordance with the principles of Good Clinical Practice and the Declaration of Helsinki. Patients were recruited from four referral centers in Spain (Barcelona), France (Marseille and Toulouse), Italy (Florence). All data were de-identified and ethical approval was waived by the local Ethics Committee of our institution in view of the retrospective nature of the study and all the procedures being performed as part of the routine care. The cohort included KT patients aged ≥ 18 years with US managed endoscopically between 2009 and 2021. Patients with missing variables required to analyze outcomes were excluded. The decision to perform endoscopic management was left to the clinical judgment of the treating physician after discussion with each patient regarding the potential benefits and side effects of all available treatment modalities for the management of US after KT.

We reviewed donor and recipient baseline characteristics, allograft description (number of arteries, veins, ureters, laterality), KT surgery variables, ureteral stenosis description (clinical presentation, initial management, length, shape, location, and onset time) and endourological management variables (the use of balloon and/or laser and complications).

The postoperative outcomes were reviewed at 6 and 12 months and at the last follow-up visit and included creatinine values and estimated glomerular filtration rate (eGFR).

Clinical failure was defined as the need for upper urinary tract catheterization, surgical repair or transplantectomy at 6, 12 and last follow-up visit. Follow-up duration was defined as the time between endoscopic procedure and the last follow-up visit.

The statistical analysis was performed using Prism software; descriptive statistics using central tendency and frequencies for continuous and categorical variables, respectively, were used. Inferential statistics were employed for the evaluation of potential predictive factors for failure, using Chi2 test and presented as risk ratios; *p* values < 0.05 were considered as statistically significant.

## Results

A total of 44 KT recipients were included. Table 1 shows the patients’ baseline characteristics. Twenty-nine (75%) KT were obtained from deceased donors, and the median age in the overall cohort was 52 years (IQR 35–63). Eight (18.2%) patients had a pyelo-ureteral anastomosis, while the rest (81.8%) had an ureterovesical anastomosis.

**Table 1:** Baseline characteristics

	*N* = 44	%
Donor		
Type of donor		
Living donor unrelated	4	9.1
Living donor related	7	15.9
Deceased donor	33	75.0
Sex		
Male	24	54.5
Female	20	45.5
Recipient		
Sex		
Male	27	61.4
Female	17	38.6
Obesity	9	20.4
BMI (median, IQR)	24.1	21.7–26.9
Hypertension	38	86.4
Diabetes mellitus	10	22.7
Charlson score (median, IQR)	5	3–5
Numer of kidney transplants		
1	40	90.9
2	2	4.5
3	2	4.5
Numer of arteries		
1	36	85.7
2	5	11.9
3	1	2.4
Number of veins		
1	41	97.6
2	1	2.4
Number of ureters		
1	41	97.6
2	1	2.4
Transplantation side		
Right	23	57.5
Left	17	42.5
Ureteral anastomosis		
Campos-Freire	3	7.3
Lich-Gregoir	26	63.4
Politano-Leadbetter	2	4.5
Pyelo-ureteral	8	18.2
Taguchi-Alferez	2	4.5
Delayed graft function	8	18.2
Nadir eGFR (Me, IQR)	41.9	(30.5–49)
BK virus infection	7	15.9

The clinical presentation of US was as acute renal failure in 31 (79.5%) cases. The median time to US onset was 3.5 months (IQR 1.9–10.8) and was ≤ 3 months in 24 (56.8%) patients. The initial drainage consisted in a nephrostomy tube in 37 (84.1%) patients, while 7 (16.7%) had a double-J stent. The median length of US assessed by antegrade pyelogram was 10 mm (IQR 7–20). In 47.7% (*n* = 21) of cases the endoscopic approach was through antegrade ureterorenoscopy (URS), followed by retrograde URS (*n* = 19, 43.2%), and in 9.1% (*n* = 4) a combined approach was used. The use of balloon dilation only was reported in 79.1% (*n* = 34) and laser incision only in 13.9% (*n* = 6) of cases, while 2 (4.7%) cases had both procedures. Intraoperative double-J catheter insertion was registered in 80% (*n* = 35) of patients.

Clavien–Dindo II complications occurred in 7.5% (*n* = 3) of cases, and 1 patient had a IIIb (descended double-J stent that required reinsertion) (Table 2). After a median follow-up of 44.6 months (IQR 30.1–100.2), clinical success was maintained in 61% (*n* = 27) of patients (Table 3). Salvage therapies included a permanent double-J catheter for periodic replacement in 6 patients (13.6%), ureteral reimplantation in 7 patients (15.9%) and transplantectomy in 4 cases (9.1%).

**Table 2:** Ureteral stenosis description and management

	*N* = 44	%*
Clinical presentation		
Incidentally at US or CT scan	8	18.2
Acute renal injury	35	79.5
Acute pyelonephritis	1	2.6
Onset time		
Early (< 3 months)	25	56.8
Late (> 3 months)	19	43.2
Initial management		
Double-J stent	7	16.7
Nephrostomy tube	37	84.1
Location of the stricture		
Pyelo-ureteral	7	15.9
Proximal	3	7.7
Middle	3	7.7
Distal/ureterovesical	31	70.5
Length (in mm) (median, IQR)	10	7–20
Shape		
Flat/concave	13	29.5
Sharp	31	70.5
Pass of contrast at initial management	32	72.7
Endoscopic approach		
Retrograde	19	43.2
Antegrade	21	47.7
Antegrade + retrograde	4	9.1
Use of balloon only	34	79.1
Use of laser only	6	13.9
Balloon + laser	2	4.7
Passage of a Couvelaire catheter	1	2.3
Intraoperative double-J stent insertion		
No passing of stent	1	2.5
Conventional double J	35	80.0
UniJ	1	2.5
Memokath	6	15.0
Clavien–Dindo complications		
II	3	7.5
IIIa	1	2.5

^*^Valid percentages due to missing data

**Table 3:** Postoperative outcomes

	6 months	12 months	Last follow-up visit
Creatinine level	154 (106–178.5)	162 (99.5–200.5)	154 (95.4–202)
eGFR	37 (28–48)	40 (23–52)	35 (17.5–45)
Success rate (%)	28 (64)	29 (66)	27 (61)

**Fig. 1:**
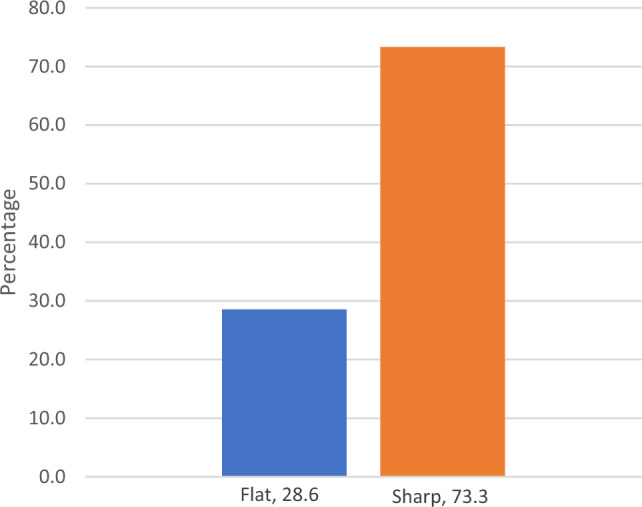
Success rate according to the shape of ureteral stenosis

**Fig. 2:**
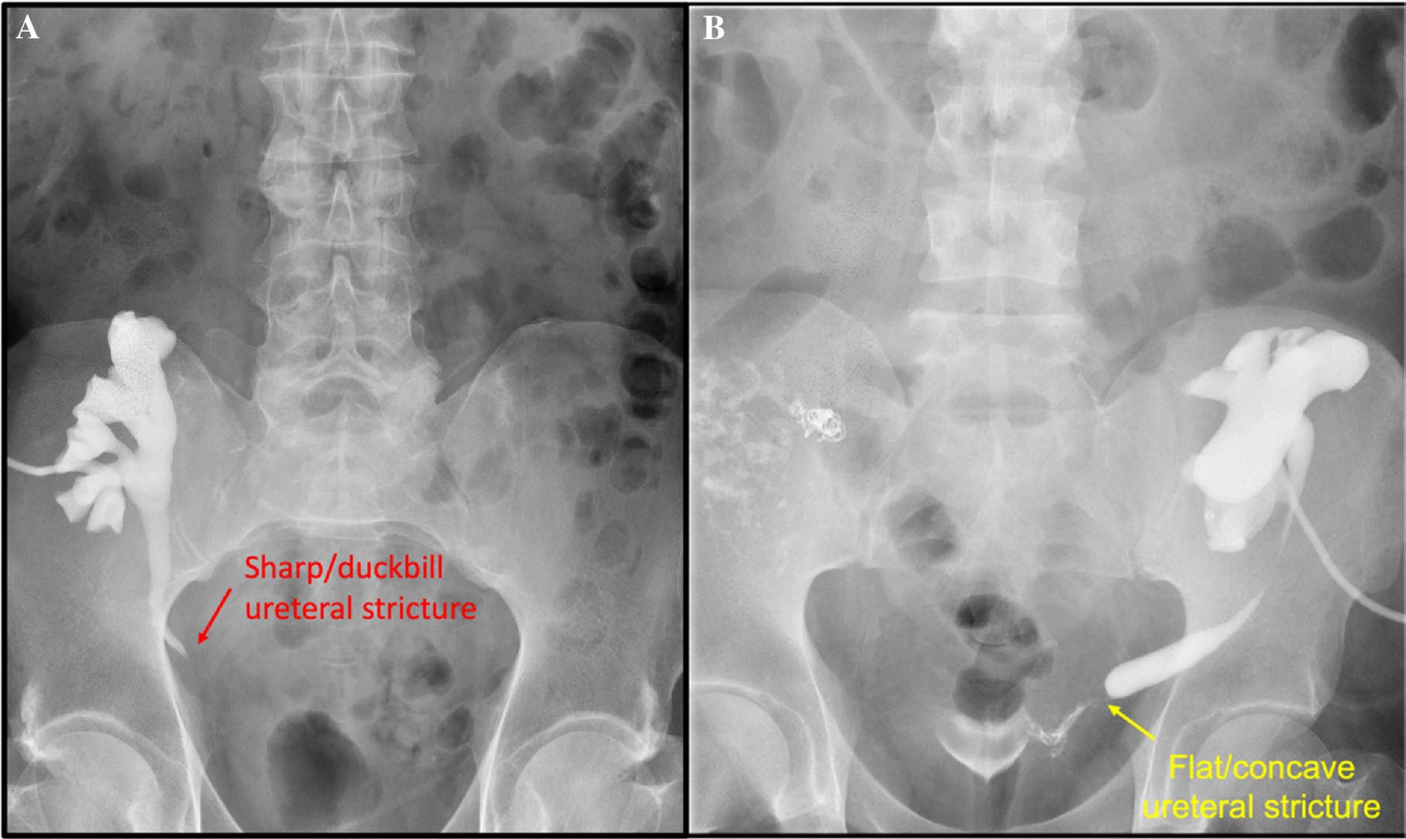
Antegrade pyelogram of ureteral stenosis. A. Sharp or duckbill shape. B. Flat or concave shape

Concerning the factors associated with treatment failure, in the bivariate analysis, having a duckbill-shaped stenosis (vs. flat/concave) was associated with treatment success (RR 0.38, *p* = 0.046, 95% CI 0.12–0.76, *p* = 0.04), while late-onset stenosis was associated with treatment failure (RR 2.00, p = 0.023, 95% CI 1.01–3.95). Figure 1 shows the success rate of endoscopic management according to the shape of US. There were no differences in treatment success regarding the location of the stenosis (*p* = 0.61). Figure 2 shows two antegrade pyelograms of both US morphologies.

## Discussion

Nowadays, choosing the optimal treatment for US in KT patients is still a matter of debate. Although minimally invasive approaches have been used for this condition for over 30 years [10], their interest has resumed with the appearance of new technologies, including the use of the different lasers available for endourology [4].

In our study, we reported a success rate of the endoscopic management of US in KT patients of 64%, 66% and 61% at 6, 12 and last follow-up visit, respectively. These rates are lower compared to the results published by Kristo et al. [2], who found a 100% success rate at a median follow-up of 24 months; however, they only included short US with a mean length of 2.8 mm while we included longer US with a median of 10 mm. Our results are similar to those of Kwong et al. [11] who reported a success rate of the endourological approach of 64.3%.

In contrast, the success rates for surgical reconstruction have been reported in previous studies to be 77% [5], which is not far from the success achieved by the endoscopic management but involves more complex strictures and cases that have failed endoscopic management (in this study, more than half of them had previously failed balloon dilation).

A recent systematic review including 34 studies and 385 patients with distal ureteral strictures after KT found success rates for surgical reconstruction as high as 85.4% in patients receiving this approach as primary treatment and as high as 93.1% as a secondary treatment, after a failed endoscopic treatment. In the same study, the endourological approach had a success rate of 64.3% and 75.5% for primary and secondary treatments, respectively, which is consistent with our results.

On the other hand, we included 6 patients who received a permanent double-J catheter, with no need for other treatments afterwards apart from periodic replacement; this approach is considered as acceptable, especially for unfit patients or those who do not wish to undergo surgery, with success rates that goes over 90% [12, 13]. Although some controversy still remains, according to previous studies, there seems to be no association between permanent ureteral stenting in KT patients and the development of complicated urinary tract infection [14–16]; therefore, we agree with other authors that this could be a reasonable solution in selected cases.

Other studies have also inquired about possible risk factors associated with the success of US endoscopic management. Gil-Sousa et al. [7] published in 2017 a single-center 10-year experience of US stenosis after KT, including both reconstructive and endoscopic treatments; they found no differences according to length of stenosis, time between transplant and stenosis or stenosis location; however, there was a clear trend towards a higher success rate in stenosis < 1.5 cm and early management < 3 months, particularly with the use of balloon dilatation, which corresponds with our results.

In line with these findings, regarding the length of the stenosis [7], He et al. [17] published a classification of US consisting of 3 grades: (1) graft function deterioration and hydronephrosis on ultrasound but no obvious stricture identified on a pyelogram; (2) focal (< 1 cm) distal ureteral stenosis at the ureteral anastomotic site, and (3) long segment (> 1 cm) distal ureteral stenosis extending to proximal ureter or pelvis. Taking this classification into account, they successfully treated 1 case as grade 1 with ureteral stent reinsertion for 6 weeks; 6 as grade 2 using balloon dilation/ureterotomy; and 5 as grade 3 with open surgery reconstruction after an initial attempt with endoscopic treatment. Their findings suggest that there may be an association between the length of the stenosis and the success of the endoscopic treatment. Interestingly, we did not find a statistically significant difference regarding the length of the US, probably because our sample of patients had a relatively short stenosis.

Another study published by Juaneda et al. [18] found that a short time to diagnosis after KT and previous acute rejection episode predicted the endourological success of a ureteral stricture. The results of these studies are similar with our findings: early-onset stenosis (< 3 months) was associated with endoscopic management success.

Currently, the EAU guidelines on KT provide recommendations on the management of US based on the timing, anatomy of the stricture (i.e., length and location), patients’ characteristics, and surgeon’s preferences [19]; however, no reference has yet been made to the shape of the stenosis and its possible implication in the success rate of endoscopically treated US.

There is a rationale from a previous study published by Gaya et al. [20] in 2023, who found that ureteroenteric anastomotic strictures were more likely to be successful with endoscopic management when these were < 1 cm (16.8% vs 4.4%, *p* < 0.001) and had a duckbill-shaped (16, 7% vs 3.1%, *p* < 0.001).

Similarly, in our study, we found that a duckbill-shaped US was associated with treatment success. This finding may have an impact on patient selection that could potentially improve postoperative outcomes. We believe further studies focusing on this aspect should be carried out to confirm our findings.

We reported few Clavien–Dindo complications (only one IIIb and 3 II cases), with no reports of Clavien–Dindo > III, which is consistent with previous published series [18].

The present study has several limitations that should be acknowledged. The main limitation lies in its retrospective design. The size of the cohort was also modest, even after including patients over a long period of time from four dedicated European KT centers, underscoring the need to increase the evidence for this management strategy to allow for greater dissemination. Having a modest sample size may confer low statistical power, which could explain why a multivariate analysis was not feasible in our study. In addition, the patients included were probably considered good candidates for these treatments beforehand, which could overestimate the success rates. Moreover, our study lacks data on the duration of balloon dilation and the pressure used, which could potentially have implications for treatment outcomes. In addition, there is a need for more data on the use of laser in these procedures.

Regardless of these limitations, we present a European multicenter study that represents one of largest series of endoscopic management of US in KT patients, including new evidence that could improve patient selection for these techniques, potentially reducing failed treatments, surgical complications, disease burden and costs.

## Conclusions

According to our study, around two-thirds of KT patients with US have a successful endoscopic management. Considering the acceptable long-term results and the safety of these procedures, we believe that the endoscopic treatment should be offered as a first-line therapy for selected KT patients with US. Those with a short and duckbill-shaped stenosis diagnosed within 3 months of KT seem to be the best candidates.

## Data Availability

The data that support the findings of this study are available from the corresponding author, upon reasonable request.

## Abbreviations

EAU: European association of urology
IQR: Interquartile range
KT: Kidney transplant
US: Ureteral stenosis
URS: Ureterorenoscopy
